# Supplementary material for: The Effectiveness of Cyrene as a Solvent in Exfoliating 2D TMDs Nanosheets
Source: Int J Mol Sci. 2023 Jun 21;24(13):10450. doi: 10.3390/ijms241310450 (PMC10342050; doi:10.3390/ijms241310450)
Supplement: Supplementary file 1 [file ijms-24-10450-s001.zip › ijms-2411494-supplementary.pdf]

Supplementary data

The Effectiveness of Cyrene as a Solvent in Exfoliating 2D TMDs Nanosheets

Jaber Adam [1,†], Manjot Singh [2,3,†], Avazbek Abduvakhidov [4], MariaRosaria Del Sorbo [5], Chiara Feoli [2], Fida Hussain [1], Jasneet Kaur [1], Antonia Mirabella [2,6], Manuela Rossi [7,8], Antonio Sasso [1], Mohammadhassan Valadan [2,3], Michela Varra [4], Giulia Rusciano [1,*] and Carlo Altucci [2,3,9,*]

[1] Department of Physics "Ettore Pancini", University of Naples "Federico II", Naples, Italy.

[2] Department of Advanced Biomedical Sciences, University of Naples Federico II, Naples,

[3] Italy National Institute of Nuclear Physics, Naples section, Naples, Italy

[4] Department of Pharmacy, University of Naples "Federico II", Naples, Italy

[5] Department of Precision Medicine, Università degli Studi della Campania "L. Vanvitelli- Naples, Italy

[6] Department of Agricultural Sciences, University of Naples Federico II, Naples, Italy

[7] Department of Earth Science, Environment and Resources, University of Naples "Federico II", Naples, Italy

[8] Istituto di Cristallografia –CNR, Via G. Amendola, 122/O 70126 Bari Italy

[9] ISASI-CNR, Institute of Applied Sciences and Intelligent Systems "Eduardo Caianiello", Naples, Italy

Correspondence- giulia.rusciano@unina.it (G.R.); carlo.altucci@unina.it (C.A.)

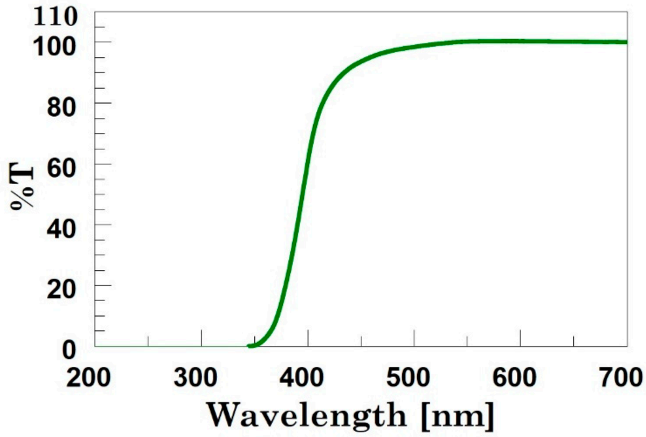

Figure S1. UV spectra of pure Cyrene (y axis is reported %Transmittance). A total extinction of the UV radiation from 200 to 360 occurs.

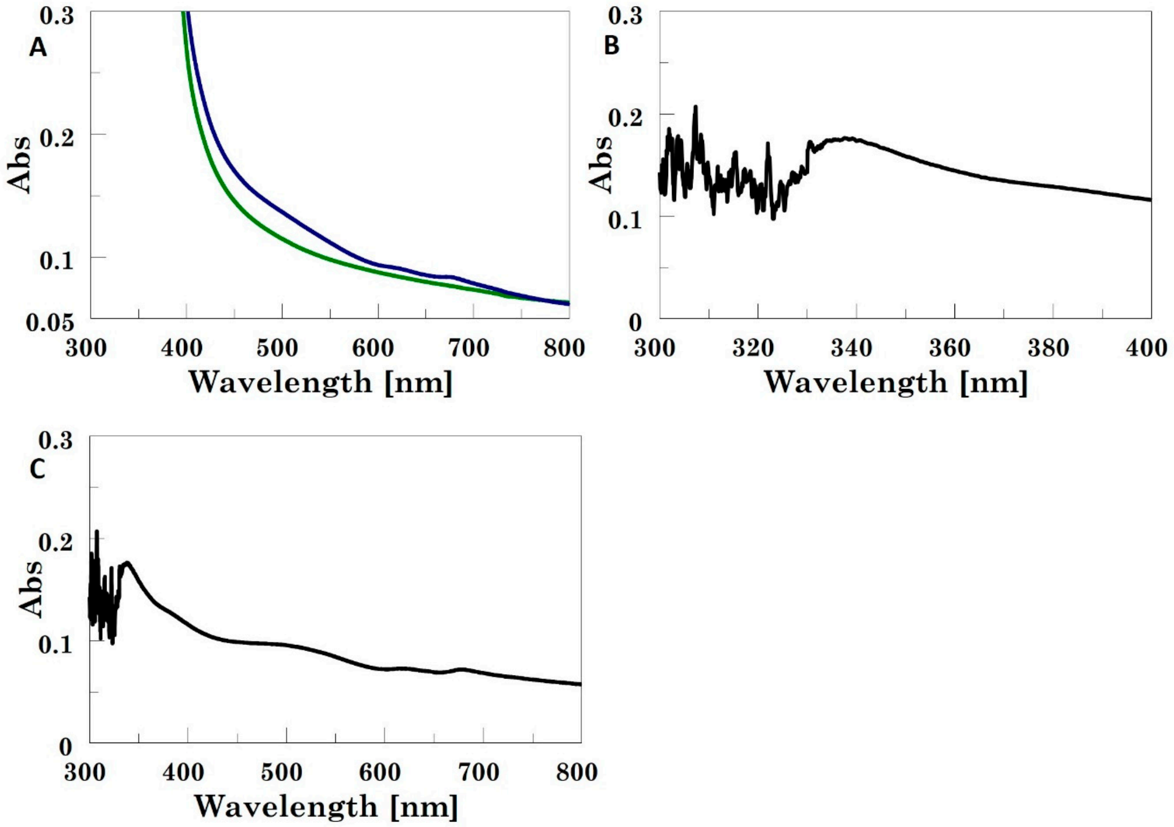

Figure S2. A. UV spectra of Cyrene: water 1:1 (green line) and MoS_2/Cyrene: water 1: 1 (blue line) **B**. Difference spectrum of MoS_2/Cyrene: water 1:1 and Cyrene: water 1:1. **C**. Enlargement of the difference spectrum in panel B. The absorbance interference of Cyrene in water did not allow to determine the minimum in the UV profile occurring in the range of 320-340 nm.

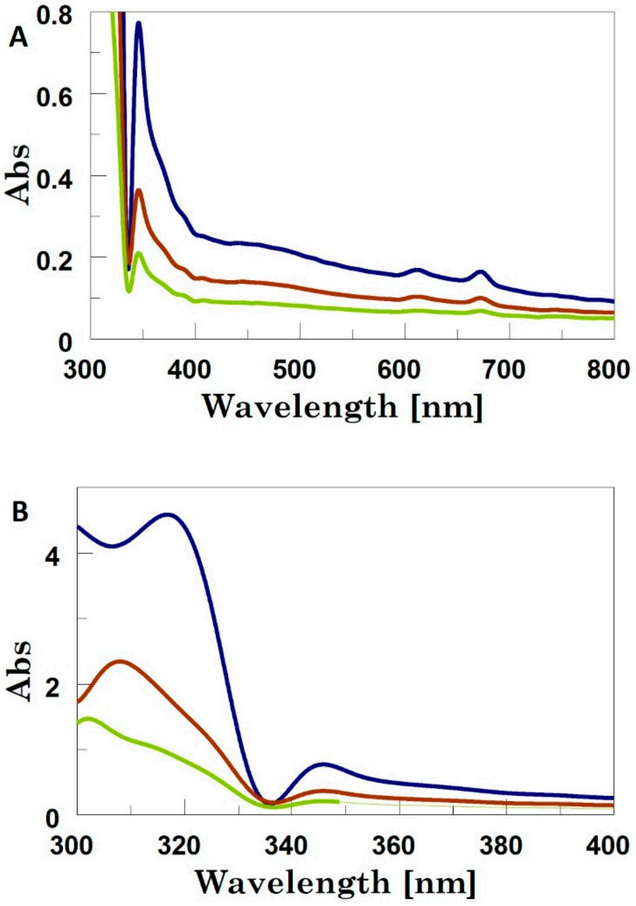

Figure S3. **A.** Deconvoluted UV-vis spectra of MoS_2/Cyrene: MeOH at dilution level 1:5 (blue line), . 1:9 (red line) and 1:17 (green line). **B**. Enlargement of the deconvoluted UV-vis spectra reported in A in the range 300-400 nm.

Table S1. Values of extinction of radiation at wavelengths corresponding to the maxima in excitons A and B and at 334 nm. The values were taken in the difference spectrum (Figure 2S)

<table>
<tr><th>2D material: Solvent</th><th>λ_A</th><th>Ext_A</th><th>λ_B</th><th>Ext_B</th><th>Ext_{334}</th></tr>
<tr><td>MoS_2/Cyrene: MeOH 1:1</td><td>669</td><td>0.310</td><td>610</td><td>0.345</td><td>0.557</td></tr>
<tr><td colspan="6">$N = 2.3x10^{36}xe^{-\frac{54888}{\lambda_A}} = 5.6$

$L(\mu m) = \frac{3.5\, Ext_B/Ext_{334} - 0.14}{11.5 - Ext_B/Ext_{334}}$</td></tr>
</table>
